# Editorial: Does Every Thyroid Cancer Patient Need Surgery?

**DOI:** 10.5041/RMMJ.10471

**Published:** 2022-04-26

**Authors:** Ziv Gil, Salem Billan

**Affiliations:** 1 The Holy Family Hospital, Nazareth, Israel; 2 Oncology Section, Rambam Health Care Campus, Haifa, Israel

In the management of malignant thyroid disorders, the standard primary treatment is thyroidectomy, a surgical resection of the thyroid gland. This procedure has been performed for over a century. Hence, it comes as no surprise that it is not only exceedingly well-described in the literature, but that a range of treatment and surgical techniques are available.^1^ However, in general, differentiated thyroid cancer is predominantly treated by two types of procedures: hemithyroidectomy and total thyroidectomy, which necessitate the complete removal of one or both of the thyroid lobes, respectively. Beyond excision of the thyroid gland, the objective of this surgery is to preserve the parathyroid glands and their blood supply, and prevent injury to the superior and recurrent laryngeal nerves.^2^

The last 30 years have seen an increasing incidence of thyroid cancer, both in early and advanced papillary thyroid cancer (PTC). This is due in part to increased diagnoses of occult thyroid cancer and exposures to environmental factors.^3^

Some thyroid cancer patients have an indolent disease course. Accordingly, many of these patients can be treated by lobectomy, without the need for radioactive iodine (RAI) ablation or thyroid-stimulating hormone (TSH) suppression. In contrast, patients with high-risk thyroid cancers often require total thyroidectomy followed by RAI ablation.^4^

Based on recent retrospective data and limited clinical trials, treatment guidelines issued by the American Thyroid Association (ATA) and the United States-based National Comprehensive Cancer Network (NCCN) recommend the option of active surveillance for low-risk differentiated thyroid cancer, which is defined as tumors smaller than 1 cm.^5^,^6^ In addition to tumor size and stage, when tailoring a treatment strategy, other patient factors should be taken into account, including age, gender, family history, and previous exposure to radiation.^7^

A paper published in this issue, by Chaturvedi et al., challenges the standard, widely practiced clinical inclination toward surgery as the first and best option for all patients with early thyroid cancer.^8^ The authors, from Mumbai’s Tata Memorial Hospital in India, are a well-established group of head and neck surgeons and oncologists and are regarded as leaders in the field. Their approach involves the use of the Surveillance, Epidemiology, and End Results (SEER) database—with records spanning over four decades (1975–2016)—to identify patients with differentiated thyroid cancers that are <4 cm (T1–2) in size with no loco-regional or distant disease (N0M0). With this resource, they identified 467 cases that received no treatment and compared their disease course against 28,261 patients who had been treated with surgery. Among the treated population, the rate of thyroid-related death was 0.3%, while the rate stood at 3.4% among those receiving no treatment. Importantly, there was no significant difference in thyroid-specific death rates between the treated and untreated subsets for T1a (*P*=0.1529) and T1b (*P*=1.000), respectively.

The authors concluded that all patients with T1 disease (tumors ≤2 cm at the greatest dimension and limited to the thyroid) have a similar prognosis whether or not they undergo surgery. The authors suggest extending the current recommendation of observation to all low-risk T1N0M0 patients, while the benefits of intervention may only be applicable for those who are more than 73 years of age with nodules greater than 26 mm.

We have previously shown that the outcomes for differentiated thyroid cancer patients whose treatments were delayed were similar to those who were operated on immediately after diagnosis.^9^ In line with our findings, the Ito et al. group suggested that observation may be a safe and prudent treatment option for low-risk thyroid cancer patients.^10^ Hence, Chaturvedi et al. offer further evidence that the pendulum may be swinging toward non-surgical strategies in the treatment of thyroid cancer. However, before any determinations are made regarding a radical change in currently accepted guidelines, one should be mindful of the limitations of their investigation.

The work of Chaturvedi et al. was based on the SEER database, which contains limited information on patients, thus making each group (surgery versus observation) non-homogeneous.^8^ For example, there is no consensus opinion presented on the radiologic evaluation of the patients, nor is an assessment offered regarding the existence of neck nodes or contralateral disease. Therefore, the TNM classification of the patients may be biased. Additionally, no clinical-demographic comparison is made between groups and no explanations given for decisions taken to perform surgery or not. Hence, those in the treatment group probably had more risk factors than the observation group. Similarly, there is no information provided on adjuvant treatment or TSH suppression, which is known to impact disease progression. Finally, treatment types (hemithyroid, total thyroid, and neck dissection) are not mentioned. For these reasons, performing a comparison of treated to untreated patients, which should be executed via multivariate analysis, is not possible.

It may well be that the Tata group study will lead to a practice change for what had long been regarded as an indolent disease. Nevertheless, before active surveillance is considered as a new standard of care for patients with low-risk T1 differentiated thyroid cancer, the hypothesis that all low-risk tumors are similar should be supported by level-1 evidence. Hence, until new data arrive, surgery should continue to be the standard of care for T1b patients.

## Abbreviations

ATA: American Thyroid Association
NCCN: National Comprehensive Cancer Network
N0M0: no loco-regional or distant disease
PTC: papillary thyroid cancer
RAI: radioactive iodine
TSH: thyroid-stimulating hormone.

## References

[1] Gil Z, Patel SG (2008). Surgery for thyroid cancer. Surg Oncol Clin N Am.

[2] Khafif A, Pivoarov A, Medina JE, Avergel A, Gil Z, Fliss DM (2006). Parathyroid hormone: a sensitive predictor of hypocalcemia following total thyroidectomy. Otolaryngol Head Neck Surg.

[3] Na’ara S, Amit M, Fridman E, Gil Z (2016). Contemporary management of recurrent nodal disease in differentiated thyroid carcinoma. Rambam Maimonides Med J.

[4] Fridman E, Gil Z (2016). What is the minimal surgery for papillary thyroid carcinoma?. Rambam Maimonides Med J.

[5] Haugen BR, Alexander EK, Bible KC (2016). 2015 American Thyroid Association management guidelines for adult patients with thyroid nodules and differentiated thyroid cancer: The American Thyroid Association Guidelines Task Force on Thyroid Nodules and Differentiated Thyroid Cancer. Thyroid.

[6] Haddad RI, Nasr C, Bischoff L (2018). NCCN guidelines insights: thyroid carcinoma, version 2. 2018. J Natl Compr Canc Netw.

[7] Na’ara S, Mahameed K, Amit M (2019). Efficacy of posttreatment radioiodine scanning in patients with differentiated thyroid cancer. Head Neck.

[8] Chaturvedi P, Singh A, Bhattachargee (2022). Defining early thyroid cancer based on population level outcomes: a need to revisit the current practice. Rambam Maimonides Med J.

[9] Amit M, Rudnicki Y, Binenbaum Y, Trejo-Leider L, Cohen JT, Gil Z (2014). Defining the outcome of patients with delayed diagnosis of differentiated thyroid cancer. Laryngoscope.

[10] Ito Y, Miyauchi A (2015). Nonoperative management of low-risk differentiated thyroid carcinoma. Curr Opin Oncol.

